# Global projections of future cropland expansion to 2050 and direct impacts on biodiversity and carbon storage

**DOI:** 10.1111/gcb.14459

**Published:** 2018-10-24

**Authors:** Amy Molotoks, Elke Stehfest, Jonathan Doelman, Fabrizio Albanito, Nuala Fitton, Terence P. Dawson, Pete Smith

**Affiliations:** ^1^ Institute of Biological and Environmental Sciences University of Aberdeen Aberdeen UK; ^2^ PBL Netherlands The Hague The Netherlands; ^3^ Department of Geography King's College London London UK

**Keywords:** biodiversity, carbon storage, cropland expansion, ecosystem services, land use change

## Abstract

Cropland expansion threatens biodiversity by driving habitat loss and impacts carbon storage through loss of biomass and soil carbon (C). There is a growing concern land‐use change (LUC) to cropland will result in a loss of ecosystem function and various ecosystem services essential for human health and well‐being. This paper examines projections of future cropland expansion from an integrated assessment model IMAGE 3.0 under a “business as usual” scenario and the direct impact on both biodiversity and C storage. By focusing on biodiversity hotspots and Alliance for Zero Extinction (AZE) sites, loss of habitat as well as potential impacts on endangered and critically endangered species are explored. With regards to C storage, the impact on both soil and vegetation standing C stocks are examined. We show that if projected trends are realized, there are likely to be severe consequences for these resources. Substantial loss of habitat in biodiversity hotspots such as Indo‐Burma, and the Philippians is expected as well as 50% of species in AZE sites losing part of their last remaining habitat. An estimated 13.7% of vegetation standing C stocks and 4.6% of soil C stocks are also projected to be lost in areas affected with Brazil and Mexico being identified as priorities in terms of both biodiversity and C losses from cropland expansion. Changes in policy to regulate projected cropland expansion, and increased measures to protect natural resources, are highly likely to be required to prevent these biodiversity and C losses in the future.

## 1 INTRODUCTION

One of the greatest challenges of the 21st century is to meet society's growing food needs whilst simultaneously reducing the environmental impact of agriculture (Foley et al., 2011). As a result of global population increase and changing demand, it is estimated that between 60%–110% more food could be needed by 2050 (Alexandratos & Bruinsma, 2012; Dawson, Perryman, & Osborne, 2016; Godfray et al., 2010; Tilman, Balzer, Hill, & Befort, 2011). Whilst increased production can partially be met through sustainable intensification on existing land (Garnett et al., 2013), substantial expansion of agriculture is expected as it is unlikely that all production increases will come from current agricultural land (Delzeit, Zabel, Meyer, & Václavík, 2017; Popp et al., 2017).

Agricultural land use already makes up one of the largest terrestrial biomes on the planet (Foley et al., 2011) yet according to the FAO, cropland is expected to expand globally by 7% until 2030 (Alexandratos & Bruinsma, 2012). However, of the world's 13.4 billion hectare land surface, only 3 billion is suitable for crop production (Bruinsma, 2003), which is restricted by availability of land resources and local natural conditions (Delzeit et al., 2017). Half of this is already cultivated (Smith et al., 2010), and although there is still a large area of land that would be highly suitable for agriculture that is not currently under cultivation (Delzeit et al., 2017), a large fraction of remaining land is currently beneath tropical forests (Smith et al., 2010). Therefore global reviews of cropland availability which exclude forests indicate there is almost no room for cropland expansion (Eitelberg, Vliet, & Verburg, 2015). The majority of current cropland expansion occurs in the tropics, with as much as 80% of new croplands replacing forests (Foley et al., 2011); however, tropical forests are especially important for C storage and for biodiversity (Johnson, Runge, Senauer, Foley, & Polasky, 2014). It is therefore extremely undesirable to convert these natural ecosystems, the consequences of which would include increased greenhouse emissions, deterioration of soil quality, degradation of land and freshwater through pollution from chemical fertilisers and loss of biodiversity (Foley et al., 2005, 2011; Smith et al., 2013).

Deforestation is estimated to account for 20% of worldwide annual C emissions (IPCC, 2007) and cleared tropical forests release ~95–215 more tonnes of C per hectare than grasslands or pastures (West et al., 2010). Forests tend to have the largest standing stock of C, which is often in the region of hundreds of tonnes of C per hectare (Albanito et al., 2016), as well as the largest inputs of C into the soil (Smith, 2008). Grasslands also tend to have large inputs into the soil, although the inputs are often less recalcitrant than forest litter (Smith, 2008). Carbon inputs to the soil are largely determined by land use (Smith, 2008), and globally, soils contain 1,500 Gt of C to one metre depth, which is twice that contained in the atmosphere (Smith, 2012) and greater than the amount in living vegetation (Post & Kwon, 2000). Although often overlooked, global soil management can contribute significantly to climate change mitigation (Paustian et al., 2016). A small percentage change in soil C can release a large quantity of C and have a substantial impact on the atmosphere (Smith, 2012). Therefore, despite high yields, food production gains from deforested land are tempered by high C losses (West et al., 2010), with deforestation rendering soil less fertile and more prone to erosion and degradation, undermining soil quality and soil health (Smith et al., 2015).

As well as being one of the largest sources of human‐induced climate change, conversion of natural ecosystems is the single most important driver of species extinctions (Baillie, Hilton‐Taylor, & Stuart, 2004). Global studies have found a strong association between C stocks and species richness (Strassburg et al., 2010) and natural habitats with greater soil C stocks are often associated with not only more species, but more threatened species (Sheil, Ladd, Silva, Laffan, & Heist, 2016). Assuming that higher than average rates of habitat loss continue, 40% of species in some of the most biologically diverse areas around the world could be lost within the next decade (Pimm & Raven, 2000). Rapid further losses are predicted under a business as usual land use scenario (Newbold et al., 2015), and it is projected that 1%–10% of the world's species will be lost in the next quarter of a century, which is a rate comparable to the Cretaceous extinction event (Chappell & LaValle, 2011). Current rates of biodiversity loss range between several hundred times the background (natural) rate (Pimm, Russell, Gittleman, & Brooks, 1995) to 1,000–10,000 times the background rate (Chappell & LaValle, 2011). This biodiversity loss raises concerns for the consequences on ecosystem functioning (Civantos, Thuiller, Maiorano, Guisan, & Araújo, 2012) and in turn, the delivery of ecosystem services, resilience of social‐ecological systems and human welfare (Corvalan, Hales, & McMichael, 2005).

Preliminary studies have identified areas of potential conflict between increased agricultural expansion and biodiversity on a regional scale (Molotoks, Kuhnert, Dawson, & Smith, 2017). However, there have been only a few global studies addressing future LUC impacts on vulnerable biodiversity and ecosystem carbon storage (Seto, Güneralp, & Hutyra, 2012), and even fewer which are spatially explicit. Furthermore, there have been global studies on the impacts of cropland expansion on carbon storage (Johnson et al., 2014; West et al., 2010) and biodiversity (Delzeit et al., 2017) separately; however, most global studies often define biodiversity as the total number of species, as opposed to focusing on habitats and species most at risk. Studies have shown a high correlation between species richness and carbon storage (Sheil et al., 2016; Strassburg et al., 2010). Therefore this study aims to examine where the direct impacts of cropland expansion are most likely to be highest for both C storage and the most vulnerable, irreplaceable areas of biodiversity.

## 2 MATERIALS AND METHODS

**Figure 1:**
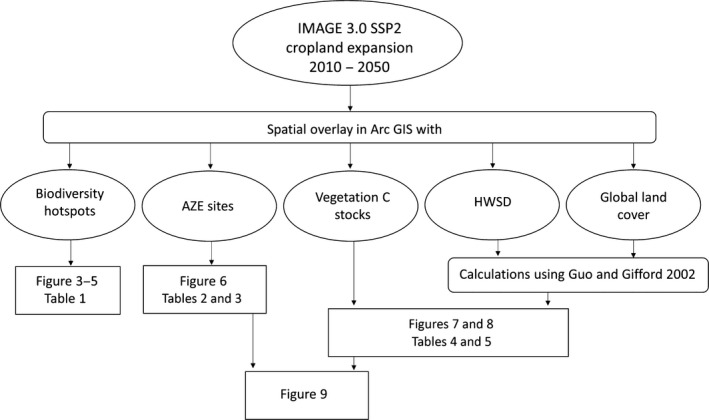
Graphic showing various steps taken in the methodology to produce results

Future cropland expansion was assessed using spatially explicit projections of change in cropland area from the integrated assessment model IMAGE 3.0 (Stehfest et al., 2014) from 2010–2050. These projections were then overlaid with various data sets using ArcGIS to demonstrate the impact of this LUC on biodiversity and C storage (Figure 1).

IMAGE 3.0 is a comprehensive integrated modelling framework, suited to large‐scale and long‐term assessments of the impacts of human activities on natural systems (Stehfest et al., 2014). The model identifies socioeconomic pathways and projects implications for energy, land, water and other natural resources (Stehfest et al., 2014). The scenario we used was the Shared Socio‐Economic Reference Pathway (SSP) 2 scenario, on a 30 × 30 arc minute resolution, showing percentage per grid cell converted to cropland over the 40 year time period. SSP2 represents the continuation of current trends with regard to development and is referred to as the “middle of the road” scenario (O’Neill et al., 2014). Social, economic and technological trends do not shift significantly from historical patterns (Popp et al., 2017) with trade tariffs and subsidies assumed to stay at current levels (Doelman et al., 2018). LUC is incompletely regulated, with tropical deforestation continuing but at slowly declining rates over time (Popp et al., 2017). Rates of crop yield increase also decline slowly with calorie consumption and animal calorie shares converging towards high levels (Popp et al., 2017). The overall regional crop yield changes are calibrated to the FAO Agricultural Outlook (Alexandratos & Bruinsma, 2012) with 50% of the improvement in agricultural efficiency being autonomous whilst the other 50% being price driven (Doelman et al., 2018). In regard to biodiversity, an assumption of the model is that protected areas are allocated as to protect 17% of each biome; however, no allocations for other important areas of biodiversity are implemented. Using this baseline scenario, we examine impacts of LUC under the presumption that no major policy changes occur and that intermediate challenges are present with respect to mitigation of, and adaptation to, climate change (O’Neill et al., 2014).

To demonstrate the impact on biodiversity through loss of habitat as a result of cropland expansion, two data sets were selected, based on fundamental principles of conservation: vulnerability and irreplaceability. The establishment of biodiversity conservation priorities is commonly addressed using this framework (Margules & Pressey, 2000). Vulnerability measures the risk to species present which are highly threatened yet are unprotected, whilst irreplaceability measures the extent to which spatial substitutes exists for securing biodiversity (Mittermeier, Turner, Larsen, Brooks, & Gascon, 2011). Areas with high levels of endemism, for example, are irreplaceable (Mittermeier et al., 2011) and prioritization of endemic species, and their habitats are crucial points for conservation actions (Bacchetta, Farris, & Pontecorvo, 2012).

Biodiversity hotspots were originally identified based on the two principles and are defined by exceptional concentrations of endemic species which were experiencing an extreme rate of habitat loss (Myers, Mittermeier, Mittermeier, Fonseca, & Kent, 2000). Therefore, we first used this independent data set of 35 existing biodiversity hotspots (Mittermeier et al., 2011, Figure 2) which have been confirmed as priority regions for the efficient conservation of biodiversity (Mittermeier et al., 2011). Each hotspot holds at least 1,500 endemic plant species and each having lost 70% or more of its original habitat extent (Mittermeier et al., 2011). A spatial overlay between these locations and projected cropland expansion to 2050 was therefore conducted at a 30 × 30 arc minute resolution to examine impact on biodiversity in terms of habitat cleared within hotspots. The percentage of each hotspot projected to be converted to cropland was calculated in ArcGIS after harmonising the spatial consistency of the data sets.

**Figure 2:**
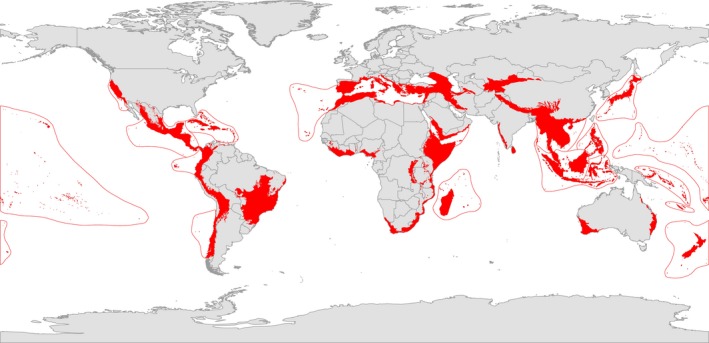
Biodiversity hotspots (red) and their outer limit (red line) (Mittermeier et al., 2011)

The conservation planning principles of irreplaceability and vulnerability are used to identify not only important habitats (Mittermeier et al., 2011) but also specific species. The Alliance for Zero Extinction (AZE) engages 88 non‐governmental biodiversity conservation organizations working to prevent species extinctions (AZE, 2010). It identifies sites where species evaluated to be Endangered or Critically Endangered under IUCN‐World Conservation Union criteria and is restricted to single remaining sites with definable boundaries, containing more than 95% of the global population (AZE, 2010; Ricketts et al., 2005). Currently, 587 sites for 920 species of mammals, birds, amphibians, reptiles, conifers and reef‐building corals have been identified with 81% AZE sites being found within a biodiversity hotspot (AZE, 2010). These species are endemic, rare and threatened (McDonald, Kareiva, & Forman, 2008) with small, restricted populations and little official protection, so are extremely vulnerable to habitat destruction (Ricketts et al., 2005). These species face extinction either because of their remaining habitat being degraded locally or because of their restricted global range making them vulnerable to external threats (AZE, 2010). We therefore decided to use this second, independent data set in another spatial overlay in ArcGIS at the same spatial resolution to examine infringement of cropland expansion on AZE sites. The sum of species per region was then calculated to estimate total species impacted by future cropland expansion.

To examine C storage loss, spatial overlays were also used. For vegetation C stocks, the cropland expansion projections were overlaid with current vegetation C stocks data, focusing on forests. Data sets for 14 individual forest types were combined using spatial joins and the resulting data set used for calculations at 1 km resolution in tonnes per hectare. The vegetation C stocks used are those presented in Ruesch and Gibbs (2008) for land covers represented in the Global Land Cover 2000 map (Arino, Ramos, Kalogirou, Defourny, & Achard, 2010). They represent the total biomass C stored in both above and below ground vegetation. Where cropland expansion projections overlapped with forests, the C stored is lost as a result of vegetation being cleared.

Soil C can also be lost as a result of LUC. To examine this, we used current soil C stocks represented in the Harmonized World Soil Database (FAO/IIASA/ISRIC/ISS‐CAS/JRC v.1.1, 2009) 30‐arc second resolution grids for each land use represented in the Global Land Cover 2009 map (Arino et al., 2010) using the total organic soil C stock density to a depth of 1 m reported by Hiederer and Kochy (2012). Together, the HWSD and Global land cover data set were overlaid with the cropland expansion projections (Figure 1). Calculations per grid cell were used to estimate C lost, using estimates from a global meta‐analysis of the impacts of LUC on soil organic C (Guo & Gifford, 2002), that is, 42% and 59% loss of SOC when converting to cropland from forest and grassland, respectively. Comparisons were then made on a regional scale, identifying areas which are projected to experience both high impacts on biodiversity as well as large losses of C storage.

## 3 RESULTS

### 3.1 Biodiversity hotspots

**Figure 3:**
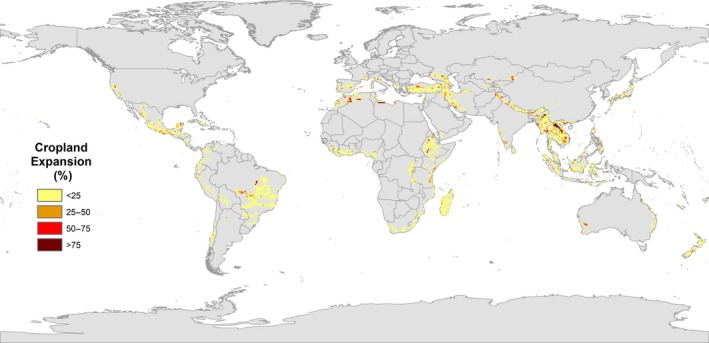
Global forecasts of cropland expansion into biodiversity hotspots from 2010 to 2050 under SSP2 as predicted by the IMAGE model

**Figure 4:**
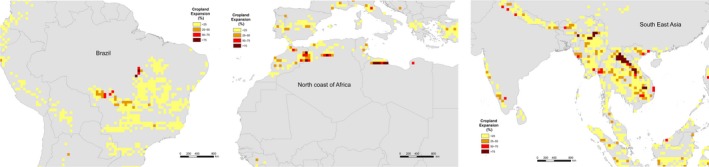
Areas affected by concentrated, high percentages of conversion of habitat to cropland

The majority of overlap of cropland expansion within biodiversity hotspots occurs in the tropics. The three main areas within hotspots affected by high percentages of conversion to cropland are the fringe of the Amazon basin in Brazil in the Cerrado hotspot, the Northern coast of Africa in the Mediterranean basin hotspot and several countries in South East Asia including Laos, Cambodia, Vietnam and Myanmar (Figures 3 and 4). These countries are located within Indo‐Burma, the most threatened hotspot, with over 7.5% of the entire area predicted to be completely converted to cropland, which is approximately almost 180,000 km^2^ (Table 1).

**Table 1:** Percentage and area of each biodiversity hotspot converted to cropland

Hotspot	Area converted (sq km)	Total hotspot extent (sq km)	Percentage converted (%)
Indo‐Burma	178,677	2,373,057	7.53
Mediterranean Basin	125,888	2,085,292	6.04
Cerrado	67,741	2,031,990	3.33
Irano‐Anatolian	59,903	899,773	6.66
Sundaland	54,264	1,501,063	3.62
Eastern Afromontane	43,479	1,017,806	4.27
Mesoamerica	38,631	1,130,019	3.42
Himalaya	37,792	741,706	5.10
Caucasus	26,776	532,658	5.03
Madagascar and the Indian Ocean Islands	24,850	600,461	4.14
Philippines	22,601	297,179	7.61
Wallacea	22,209	338,494	6.56
Guinean Forests of West Africa	21,820	620,314	3.52
Atlantic Forest	18,741	1,233,875	1.52
Madrean Pine‐Oak Woodlands	16,083	461,265	3.49
Japan	15,434	373,490	4.13
New Zealand	14,150	270,197	5.24
Mountains of Central Asia	13,740	863,362	1.59
Coastal Forests of Eastern Africa	12,550	291,250	4.31
Tropical Andes	10,301	1,542,644	0.67
Southwest Australia	9,157	356,717	2.57
Western Ghats and Sri Lanka	9,104	189,611	4.80
Horn of Africa	7,740	1,659,363	0.47
California Floristic Province	7,612	293,804	2.59
Forests of East Australia	7,355	253,200	2.90
Maputaland‐Pondoland‐Albany	6,104	274,136	2.23
Chilean Winter Rainfall and Valdivian Forests	5,977	397,142	1.51
Tumbes‐Choco‐Magdalena	3,525	274,597	1.28
Caribbean Islands	2,393	229,549	1.04
Mountains of Southwest China	2,314	262,446	0.88
Succulent Karoo	2,065	102,691	2.01
Cape Floristic Region	1,721	78,555	2.19
Polynesia‐Micronesia	1,050	47,239	2.22
East Melanesian Islands	1,011	99,384	1.02
New Caledonia	675	18,972	3.56
Total	893,436	23,743,301	3.76

**Figure 5:**
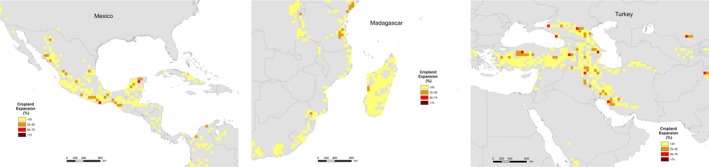
Areas affected by widespread, low percentages of conversion to cropland

Other areas, for example, Mexico, Madagascar and Turkey are affected more by relatively low‐density expansion of cropland spread across a large area (Figures 3 and 5). Turkey is located within the Irano‐Anatolian hotspot which is projected to experience the third highest percentage of loss, with over 6% of the total area being converted, which is approximately 60,000 km^2^. In contrast to Indo‐Burma, this hotspot is predicted to see widespread conversion to cropland at lower density (Figure 5).

Similarly, Madagascar and Mexico are predicted to experience low‐density cropland expansion across the entire country (Figure 5). Madagascar in particular is projected to experience cropland conversion almost country‐wide (Figure 5) even though only 4% or 24,850 km^2^ is projected to be converted to cropland (Table 1).

Indo‐Burma has the largest area converted to cropland as well as the second highest percentage of the hotspot converted at 7.53% (Table 1). The highest percentage of a hotspot being converted does not, however, necessarily equate to the largest absolute area since the total extent of each hotspot differs. For example, the Philippians hotspot has the largest percentage converted at 7.61%. However, only a total of 22,601 km^2^ converted to cropland (Table 1). Similarly, the second largest area within a hotspot projected to be converted is the Mediterranean Basin, with 125,888 km^2^ which is only 6.04% of the hotspot, followed by the Cerrado with 67,741 km^2^ or 3.33% (Table 1).

### 3.2 AZE sites

**Figure 6:**
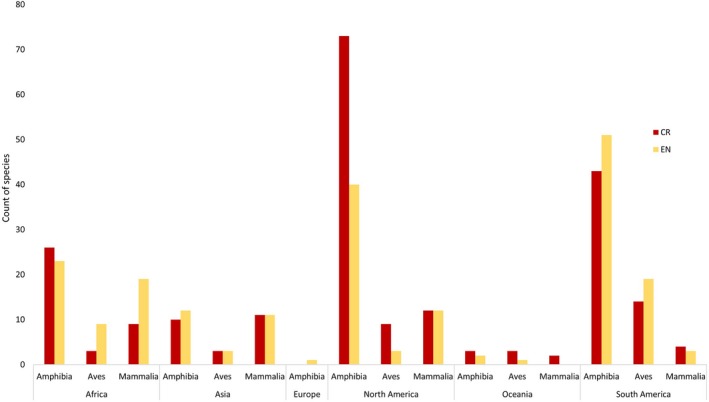
Count of all species for Critically Endangered (CR) and Endangered (EN) status in AZE sites for classes most affected by cropland expansion (Amphibia, Aves and Mammalia)

Many AZE sites are projected to experience habitat destruction as a direct result of conversion to cropland. Almost 50% of all species, 455 out of 920 (Table 2), are projected to experience loss of habitat as a direct result of this LUC. This includes almost 300 amphibian species, as well as 83 species of mammal and 67 species of bird (Table 2, Figure 6), all of which are already listed as either endangered or critically endangered by the International Union for Conservation of Nature.

**Table 2:** Count of all species in AZE sites affected by cropland expansion

Region and class affected	CR	EN	Total
Africa	41	52	93
Amphibia	26	23	49
Aves	3	9	12
Mammalia	9	19	28
Pinopsida	2	1	3
Reptilia	1	0	1
Asia	27	28	55
Amphibia	10	12	22
Aves	3	3	6
Mammalia	11	11	22
Pinopsida	1	1	2
Reptilia	2	1	3
Europe	0	1	1
Amphibia	0	1	1
North America	97	58	155
Amphibia	73	40	113
Anthozoa	1	0	1
Aves	9	3	12
Mammalia	12	12	24
Pinopsida	1	2	3
Reptilia	1	1	2
Oceania	12	4	16
Amphibia	3	2	5
Aves	3	1	4
Mammalia	2	0	2
Pinopsida	4	1	5
South America	62	73	135
Amphibia	43	51	94
Aves	14	19	33
Mammalia	4	3	7
Reptilia	1	0	1
Grand Total	239	216	455

Approximately equal numbers of critically endangered and endangered species are projected to be affected, with 240 critically endangered and 217 endangered species (Table 2). The Americas are predicted to have the largest numbers of all species affected, with the habitat of 290 species being encroached upon by future cropland expansion, followed by Africa and Asia, with 93 and 55 species being threatened, respectively (Table 2, Figure 6). In contrast, there are only 16 species affected in Oceania and a single species of amphibian affected in Europe (Table 2).

Only a small number of species are shown to be present in areas of high conversion to cropland, with only one species of amphibian in Oceania affected by the top quartile of cropland expansion (Table 3). This species may, therefore, be at higher risk than species in areas of low conversion to cropland, as the area is projected to experience a larger percentage of conversion to cropland within the AZE site. On a country level, Mexico is projected to have the highest number of AZE sites impacted by cropland expansion with 111 sites set to experience some level of conversion (Supporting information Table S1).

**Table 3:** Count of all species in AZE sites affected by each quartile of cropland expansion shown by region and class

Distinct count of species affected shown by region and class	Intensity of cropland expansion affecting AZE site (%)[Link]				Total
	<25	25–50	50–75	>75	
Africa	92	8	3	0	93
Amphibia	49	6	1	0	49
Aves	12	0	2	0	12
Mammalia	27	2	0	0	28
Pinopsida	3	0	0	0	3
Reptilia	1	0	0	0	1
Asia	51	6	1	0	55
Amphibia	22	0	0	0	22
Aves	5	1	0	0	6
Mammalia	20	3	1	0	22
Pinopsida	2	1	0	0	2
Reptilia	2	1	0	0	3
Europe	1	0	0	0	1
Amphibia	1	0	0	0	1
North America	145	29	0	0	155
Amphibia	107	20	0	0	113
Anthozoa	1	0	0	0	1
Aves	8	4	0	0	12
Mammalia	24	4	0	0	24
Pinopsida	3	0	0	0	3
Reptilia	2	1	0	0	2
Oceania	16	3	0	1	16
Amphibia	5	2	0	1	5
Aves	4	0	0	0	4
Mammalia	2	0	0	0	2
Pinopsida	5	1	0	0	5
South America	134	15	0	0	135
Amphibia	94	8	0	0	94
Aves	32	6	0	0	33
Mammalia	7	1	0	0	7
Reptilia	1	0	0	0	1
Grand Total	439	61	4	1	455

Totals do not always sum each row as each species may be affected by more than one area of cropland expansion.

### 3.3 Carbon storage

**Figure 7:**
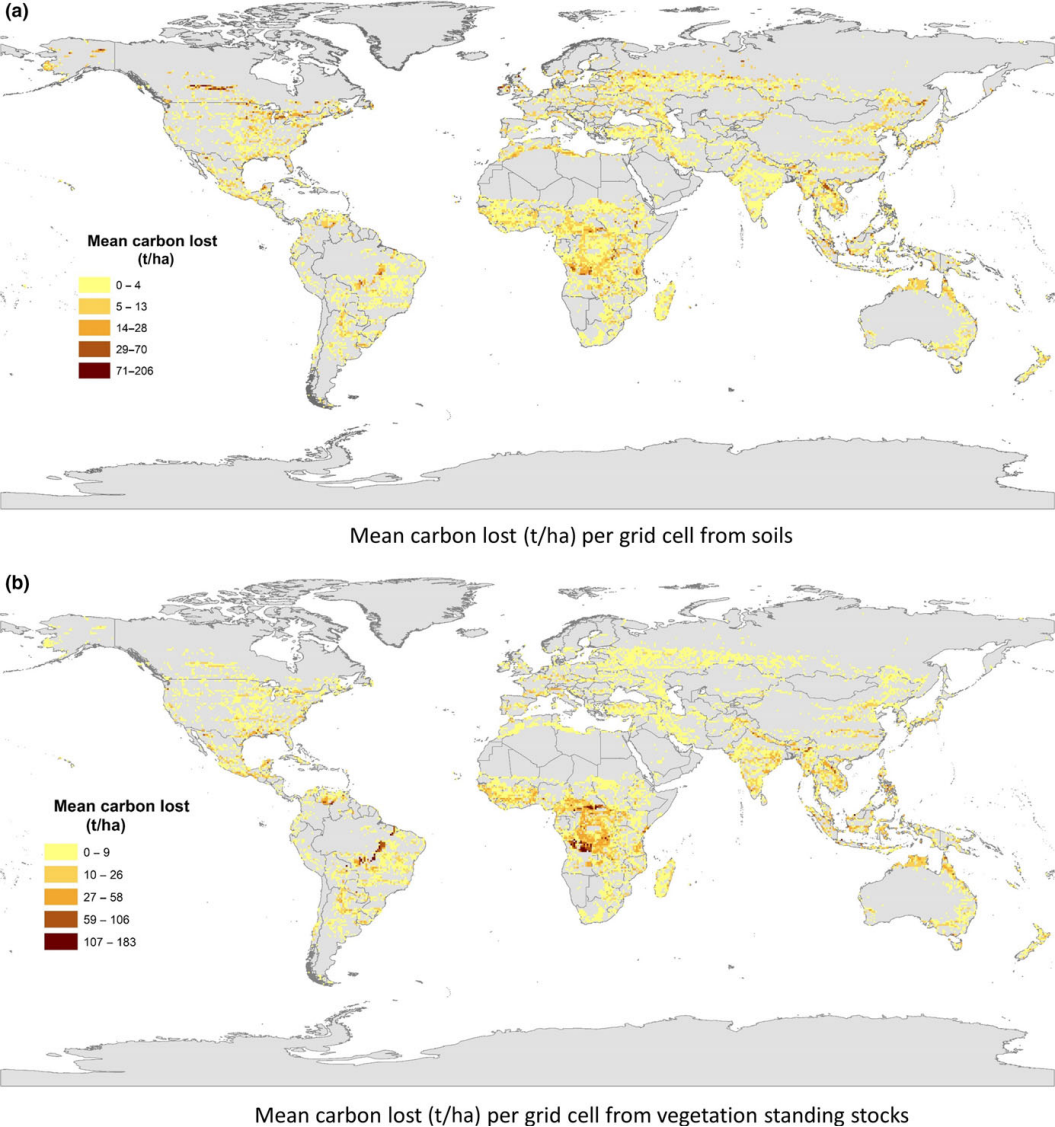
Panel showing spatial patterns of carbon lost a) from soil and b) from vegetation biomass

**Figure 8:**
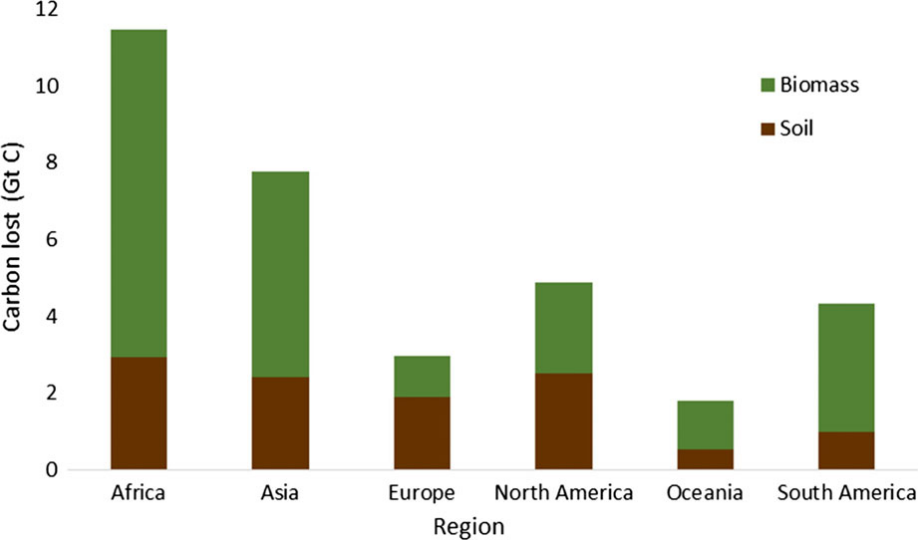
Estimated sum of carbon lost from soil and vegetation biomass per region (Gt C = billions of tonnes C)

For both soil C and vegetation biomass, Africa is projected to lose the most C storage (Figures 7 and 8), followed by Asia as a result of cropland expansion with 11.48 and 7.78 Gt C lost in each, respectively (Figure 8).

On a country level, the Democratic Republic of the Congo is estimated to lose the most C with a total of 3.19 Gt C from soil and biomass, followed by Brazil and the United States with 2.62 and 2.48Gt C estimated to be lost, respectively (Tables 4 and 5). For soil C, the United States is projected to lose the most, with an estimated loss of 1.18 Gt C followed by Russia and Canada (Table 4). For biomass C stocks in vegetation, the Democratic Republic of the Congo, followed by Brazil and Angola are estimated the experience the greatest C losses (Table 5).

**Table 4:** Top ten countries with the largest estimated soil carbon loss (Gt C = billions of tonnes C)

Country	Gt C lost
United States	1.18
Russia	1.12
Canada	1.04
China	0.56
Democratic Republic of the Congo	0.53
Brazil	0.49
Australia	0.44
Indonesia	0.43
Angola	0.34
Mexico	0.27

**Table 5:** Top ten countries with the largest estimated carbon storage loss from vegetation (Gt C = billions of tonnes C)

Country	Gt C lost
Democratic Republic of the Congo	2.66
Brazil	2.13
Angola	1.52
United States	1.30
India	1.24
Australia	1.03
Central African Republic	1.03
Indonesia	1.00
China	0.75
Mexico	0.63

**Figure 9:**
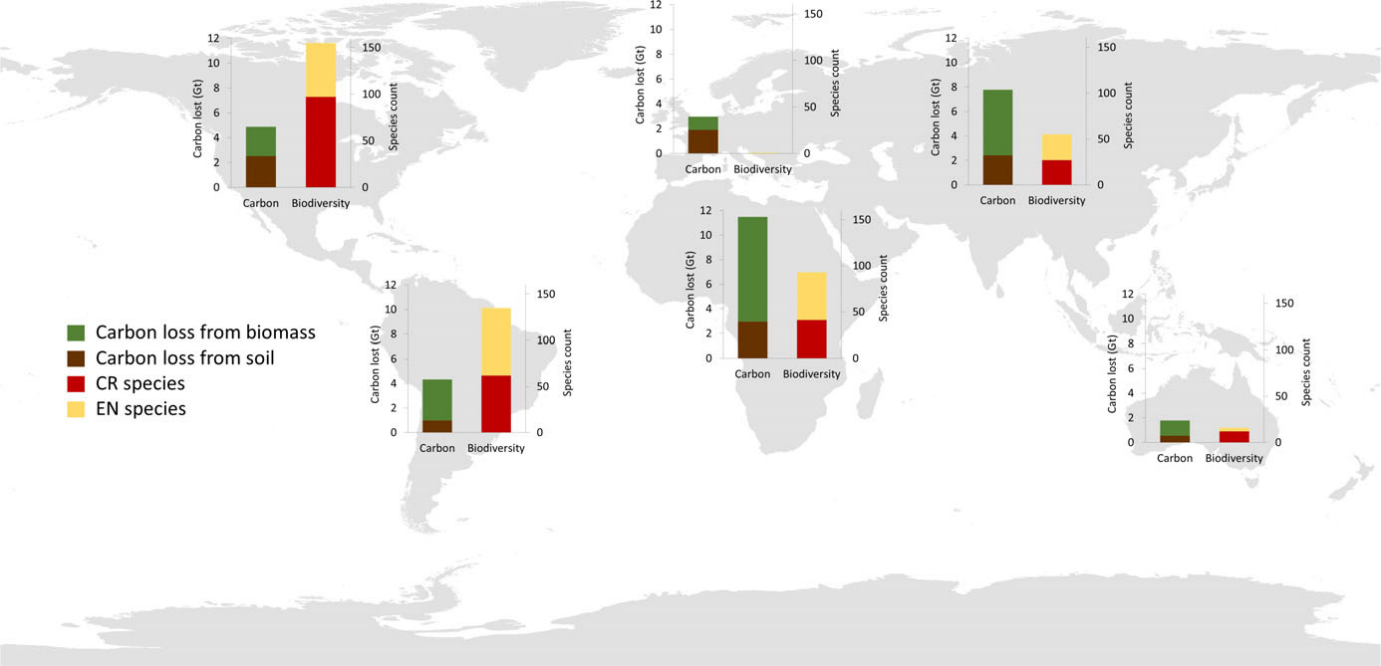
Comparison of impacts of cropland expansion for each region on carbon storage for both soil and standing vegetation stocks and AZE species, Endangered (EN) and Critically Endangered (CR)

When considering carbon and biodiversity losses together, the Americas have the most AZE species affected, but carbon losses, in comparison, are relatively small. In contrast, Asia shows large carbon losses but fewer species are impacted (Figure 9).

Africa shows the highest C losses as well as high numbers of AZE species threatened by cropland expansion. Although Madagascar is the country with most species affected, Tanzania is the only country in Africa which also has high C losses. Furthermore, on a country level, Indonesia and Mexico both have very high numbers of species at risk as well as being in the top ten countries for both soil and vegetation biomass C loss (Supporting information Table S1,).

## 4 DISCUSSION

The projections show large areas of biodiversity hotspots potentially at risk from future cropland expansion. Biodiversity hotspots cover over 23million km^2^ of which an estimated 0.9 million km^2^, approximately 3.76%, are projected to be converted to cropland by 2050 (Table 1). Although this seems a small percentage, collectively they hold over 50% of the world's endemic plant species and 77% of terrestrial vertebrates (Mittermeier et al., 2011), and 88% of the original extent of their primary vegetation land cover has already been destroyed (Myers et al., 2000). Therefore, considering the vulnerability and irreplaceability of these areas, land conversion could have profound impacts on biodiversity. Furthermore, 50% of all AZE species are estimated to be impacted through habitat destruction from this LUC (Table 2). In total, 455 species have been identified as being at risk from future cropland expansion, of which almost 300 are amphibian species (Table 2, Figure 5). Considering that AZE sites are designated based on a species which is already endangered or critically endangered, as well as being restricted to single remaining sites, these species are particularly vulnerable to external threats (AZE, 2010).

This LUC also has profound impacts on C storage with 13.7% of standing biomass C stocks and 4.6% of soil C stocks being lost in areas projected to experience cropland expansion. A total of 33 Gt C is estimated to be lost as a direct result of clearing land for cropland, with 11.3Gt lost from soil and 21.9Gt C from vegetation biomass C stocks in forests (Figure 7). Globally, forests are estimated to contain 816 Gt C (Lal, 2008) so this represents a loss of 2.67% of global C. To put this in context, in 2010 total global annual GHG emissions were estimated at 49.5 Gt CO_2_eq (= 13.5 Gt C eq.) with CO_2_ comprising over 75% which is 38 GtCO_2_ per year (= 10.4 Gt C eq.) (IPCC, 2014). The projected C loss is equivalent to therefore 2.4 times greater than the annual global anthropogenic GHG emissions or 2.9 times greater than the annual global C emissions. This loss of C stocks is therefore substantial and would contribute significantly to greenhouse gas emissions during a period when it is essential to minimize such emissions (Smith et al., 2016). Furthermore, loss of C storage in the soil often impacts supporting services such as soil formation, nutrient cycling and water quality, affecting the fertility, quality and health of the soil (Smith et al., 2015). This could in turn negatively impact biodiversity as well as the productivity of the soils newly converted to croplands.

Although biodiversity and C storage are rarely addressed together, natural habitats with greater soil C stocks have been found to often be associated with more species of conservation significance (Sheil et al., 2016; Strassburg et al., 2010). Maintaining vegetation not only protects habitat for biodiversity but also benefits water quality and maintains landscape connectivity, which contribute to supporting wildlife (Sheil et al., 2016). On a regional scale, Africa experiences the highest impact on both biodiversity and C storage. Projections estimate high C losses, especially surrounding the Congo Basin (Figure 7), which is reflected in the Democratic Republic of the Congo being one of the countries with the highest C losses (Tables 4 and 5). It also shows high numbers of AZE species impacted by cropland expansion (Figure 9). However, the Americas show low C losses compared to higher numbers of species impacted, whilst the reverse is true for Asia (Figure 9), despite having the highest losses from biodiversity hotspots with over 350,000 km^2^ converted to cropland (Table 1).

The most threatened hotspot with the largest percentage area converted to cropland is also located in Asia. Indo‐Burma is the hotspot with the largest areas of conversion to cropland, concentrated in Laos (Figures 3 and 4, Table 1). It also has the second highest percentage of total area within the hotspot lost with 7.5% being converted to cropland (Table 1). Another study also projects that Indo‐Burma will lose an additional 20% of its primary vegetation from 2005–2100 in all climate scenarios, which is the most amongst all the biodiversity hotspots (Jantz et al., 2015).

Over the past couple of decades, tropical Asia has seen unprecedented LUC and has experienced the highest deforestation rate globally (Achard et al., 2002; Tao et al., 2013) and faster cropland expansion over the past 20 years than any other region (Tao et al., 2013). Asia is characterized by faster than global average population growth, with a consequent increase in food production to meet demand, by expansion of agricultural land (Cervarich et al., 2016). Furthermore, the hotspots are home to a disproportionate share of people, with populations in hotspots growing faster than the rest of the world, and also having a substantial fraction of the world's poor (Mittermeier et al., 2011). Geographically, tropical Asia occupies one of the largest areas of tropical forests (Cervarich et al., 2016) and has relied heavily on clearing intact forests for new agricultural land (Gibbs et al., 2010). It is also likely to undergo further rapid development in the future with large areas of cropland expansion and natural forest shrinkage occurring to meet growing demands (Tao et al., 2013). It is therefore unsurprising that tropical Asia is also predicted to experience high losses of C (Figure 9) with a combined total of 1.03 Gt C lost from Laos and Myanmar alone.

Although Asia has the highest projected rates of habitat loss from conversion to cropland and large C losses, it has lower AZE species impacted (Figure 9) which is potentially because of the majority of these sites being situated in the Americas. Several countries in South America have high numbers of species impacted, for example, 38 in Colombia and 29 in Peru (supporting information Table S1). However, the numbers impacted in Mexico exceed even these, with 111 species affected out of a total of 155 in the whole of North America (Supporting information Table S1). This is not only the highest number of AZE species globally but is also more than three times the amount of threatened species affected than in any other country (Supporting information Table S1). Only a third of AZE sites are legally protected (Ricketts et al., 2005) and many are also surrounded by intense human development, placing these sites under significant risk from future LUC (Ricketts et al., 2005; Seto et al., 2012). Furthermore, Mexico also incurs heavy C losses (Tables 4 and 5) and spans across all three biodiversity hotspots found in North America, so is one of few countries projected to be heavily impacted by future cropland expansion for both vulnerable biodiversity and C storage.

Latin America has the planet's largest land reserves for agriculture (Graesser, Aide, Grau, & Ramankutty, 2015), and Mexico has been found to have high natural expansion potential as it is characterized by fertile soils and adequate climate conditions for crop growth (Delzeit et al., 2017). It is one of the most biodiverse countries in the world, with approximately 30,000 species of plants and 449 mammal species (Cantu, Wright, Scott, & Strand, 2004). However, it is also amongst the countries predicted to have the most species suffering large habitat declines by 2050 (Visconti et al., 2011, Supporting information Table S1) as most new agricultural land in Latin America has also come from intact, undisturbed forests (Gibbs et al., 2010). This is a result of large increases in food production and consumption, driven by accelerated growth of population and consumption (Visconti et al., 2011).

The majority of species projected to be affected in Mexico are amphibians, making up 83 of the 111 species (Supporting information Table S1). Likewise, on a global scale, amphibians are the most heavily impacted with 278 of the 455 AZE species impacted (Table 3). Amphibians are currently undergoing worldwide population declines which are unprecedented (Stuart et al., 2004), with habitat loss and fragmentation the main causes of this conservation crisis (Cushman, 2006). They are more threatened and are declining more rapidly than other classes such as birds or mammals and many are on the brink of extinction, with 427 species listed as critically endangered (Stuart et al., 2004). Their vulnerability in comparison to other classes can be explained by a number of factors, including low mobility, narrow habitat and climate tolerances, high susceptibility to pathogens and sensitivity to environmental pollution (Cushman, 2006). Climate change in particular has been proposed as a significant threat to amphibians, with shifts in temperatures increasing the likelihood of pathogen outbreaks (Pounds et al., 2006). It is therefore unsurprising that amphibians are shown to be the most affected class by cropland expansion into AZE sites.

Not all AZE sites are located within biodiversity hotspots, and Brazil is an example of a very biodiverse country (Cantu et al., 2004), yet has low numbers of AZE species impacted by cropland expansion (Supporting information Table S1). The Cerrado biodiversity hotspot, however, has the third largest area of land converted to cropland, with almost 70,000 km^2^ projected to be lost from this hotspot (Table 1). Brazil has, until recently, experienced the world's highest rates of tropical deforestation (Lapola et al., 2014), and as in tropical Asia, lower production costs and fewer environmental regulations have created rapid responses to increased demand for crops (Gibbs et al., 2010). Brazil is also projected to incur the second largest C losses with a combined loss of 2.62Gt C (Tables 4 and 5) and is therefore also a potential global priority in terms of future impacts on both biodiversity and C storage.

Projections from this study suggest the areas around the southern extent of the Amazon in Brazil will be particularly affected, with this “arc of deforestation” stated to be one of the most active land use frontiers in the world in terms of total forest loss (Morton et al., 2006). Approximately 62% of the forests of Amazonia are located in Brazil, with clearance concentrated on the southern and eastern margins (Malhi et al., 2008), with one of the main drivers of clearance being from mass soybean production (Gibbs et al., 2010; Malhi et al., 2008; Morton et al., 2006). The projections suggest cropland expansion in this area is likely to increase over the next 30 years, with higher rates of land conversion in small areas on the fringes of the Amazon and more widespread conversion of land at lower percentages to cropland in Southern Brazil. This is consistent with other studies showing that although deforestation rates in the Amazon have declined, cropland expansion continues in the Cerrado (Graesser et al., 2015).

Carbon losses in Brazil are second only to the Democratic Republic of the Congo, which is projected to lose 3.19 Gt C (Tables 4 and 5). A UN report examining the potential for cropland expansion concluded that Africa had more under‐utilized arable land than any other continent (Deininger & Byerlee, 2011). More than half the uncultivated area left which is unforested and unprotected is located in ten countries, six of which (Sudan, The Democratic Republic of the Congo, Mozambique, Madagascar, Chad, Zambia) are in Africa, which inevitably leaves the region at a higher risk of C loss from expansion of agriculture. Furthermore, the largest areas of forest cover are located in the Congo basin, half of which are within the boundaries of the Democratic Republic of the Congo (Potapov et al., 2012). Compared to forests in other regions, most tropical forests generally have relatively high soil C density; therefore, land conversion will significantly reduce C storage (Tao et al., 2013). Furthermore, the DRC has the highest area of annual forest cover loss because of high population density and the highest population growth rate in the region, both of which are key drivers of LUC (Potapov et al., 2012).

Although there is not a great threat to vulnerable biodiversity in this region, it is possible that clearing forests also has a significant impact on biodiversity metrics not considered in this study. This is also the case for other countries, such as the United States which appears in the top five for both categories of C loss (Tables 4 and 5), yet has very few AZE species affected (Supporting information Table S1) and even less important habitat at risk (Figure 2). The Irano‐Anatolian biodiversity hotspot, on the other hand, is also heavily impacted by cropland expansion, yet only one AZE species is located in the area (Table 2) and is also not predicted to experience large C losses. It is impossible to say what the impact on biodiversity apart from the metrics used in this study are, however the importance of these results is to highlight areas where the most vulnerable, irreplaceable species are at the greatest risk as well as heavy losses of C storage being incurred from cropland expansion.

A number of countries and regions are identified as particular priorities for careful management and regulation (or prevention) of cropland expansion for reasons of both biodiversity conservation and C storage. This includes the Indo‐Burma biodiversity hotspot in tropical Asia, as well as Mexico and Brazil, both countries being long recognized for their biodiversity and classified as mega‐diverse countries (Mittermeier, Robles‐Gil, & Mittermeier, 1997). For biodiversity alone, the Irano‐Antaolian and the Mediterranean Basin also show substantial loss of habitat, whilst for C storage alone the Democratic Republic of Congo shows the heaviest losses. However, areas where both vulnerable biodiversity and C storage are threatened perhaps deserve special attention. Having identified the areas and countries with particular vulnerability to future cropland expansion, further in‐depth research is needed within these countries to pinpoint local areas at the highest risk from cropland expansion.

Future cropland expansion may well contribute towards improved food security, but as we have demonstrated, it frequently presents a trade‐off with biodiversity (Delzeit et al., 2017) and/or carbon storage (Smith et al., 2013; West et al., 2010). Further, we have demonstrated that whilst cropland expansion is likely to lead to both biodiversity loss and ecosystem C loss, cropland expansion does not always threaten habitat, species and C storage to the same extent. As a provisioning ecosystem service, food production often has trade‐offs with almost all other ecosystem services (Raudsepp‐Hearne, Peterson, & Bennett, 2010), some of which have been shown to decline as a direct result of economic growth and enhancement of food provision (Dearing et al., 2012). Cropland expansion, as projected in this study, leads to direct impacts on both C storage and biodiversity, showing significant loss of habitat and biomass from global biodiversity hotspots, AZE sites and C pools. There is a danger that this could create positive feedback, where cropland expansion into a certain area could have repercussions on neighbouring areas and lead to further losses of other ecosystem services. However, further research is required using finer resolution data within countries to identify local areas most at risk of these repercussions. Though the full extent of the impacts of cropland expansion in these areas cannot be assessed in a global study such as this, it highlights areas particularly at risk, shows regions in which conservation policy is likely to be needed to further protect biodiversity and ensure minimal losses of C storage in these vulnerable areas, and highlights the magnitude of the threats to biodiversity and C storage posed by cropland expansion in the future if current trends are continued.
