# Supplementary material for: Global projections of future cropland expansion to 2050 and direct impacts on biodiversity and carbon storage
Source: Glob Chang Biol. 2018 Oct 24;24(12):5895–908. doi: 10.1111/gcb.14459 (PMC6282572; doi:10.1111/gcb.14459)
Supplement: Supplementary file 1 [file GCB-24-5895-s001.pdf]

Supporting information

Table S1- Count of species per country and percentage of cropland expansion effecting AZE sites

Count of species	<25	25-50	50-75	>75	Grand Total
**Africa**	**92**	**8**	**3**		**93**
Angola	2				2
Cameroon	18	1			18
Congo, The Democratic Republic of	5		3		5
Cote d'Ivoire	2				2
Cote d'Ivoire, Guinea, Liberia	3				3
Ethiopia	9				9
Kenya	2	1			3
Madagascar	20				20
Malawi	2				2
R ├®union (to France)	2				2
Rwanda	1				1
Somalia	1				1
South Africa	1				1
Tanzania, United Republic of	21	6			21
Uganda	1				1
Uganda, Kenya	2				2
**Asia**	**51**	**6**	**1**		**55**
Afghanistan	1				1
Armenia	1				1
China	6	2	1		8
India	10	2			12
Indonesia	17	2			17
Japan	3				3
Myanmar	1				1
Philippines	2				2
Sri Lanka	10				10
Vietnam	1				1
**Europe**	**1**				**1**
Turkey	1				1
**North America**	**145**	**29**			**155**
Costa Rica	1				1
Cuba	10				10
Guatemala	9				9
Honduras	12				12
Mexico	106	24			111
Panama	1				1
Trinidad and Tobago	2				2
United States of America	4	5			9

**Oceania**	**16**	**3**		**1**	**16**
Australia	7	3		1	7
Fiji	1				1
New Caledonia	4				4
New Zealand	2				2
Papua New Guinea	1				1
Samoa	1				1
**South America**	**134**	**15**			**135**
Argentina	5				5
Bolivia	7				7
Brazil	7	1			7
Chile	3				3
Colombia	36	13			36
Colombia, Venezuela	2				2
Ecuador	22				22
Peru	29				29
Venezuela	23	1			24
**Grand Total**	**439**	**61**	**4**	**1**	**455**

**Totals do not always sum each row as each species may be affected by more than one area of cropland expansion*
